# Histone Deacetylase Inhibition Enhances AQP3 Levels in Human Corneal Epithelial Cells and Corneal Wound Healing in Normoglycemic and Diabetic Male Mice

**DOI:** 10.3390/cells14231880

**Published:** 2025-11-27

**Authors:** Samuel Melnyk, Xiaowen Lu, Victoria Ronderos, Vivek Choudhary, Maribeth H. Johnson, Mitchell A. Watsky, Wendy B. Bollag

**Affiliations:** 1 Department of Physiology, Medical College of Georgia at Augusta University, Augusta, GA 30912, USA; smelnyk@augusta.edu (S.M.); victoriarc@sanjuanbautista.edu (V.R.); vivekchoudharyxyz@gmail.com (V.C.); 2 Department of Cellular Biology and Anatomy, Medical College of Georgia at Augusta University, Augusta, GA 30912, USA; xialu@augusta.edu (X.L.); mwatsky@augusta.edu (M.A.W.); 3 Department of Neuroscience and Regenerative Medicine, Medical College of Georgia at Augusta University, Augusta, GA 30912, USA; majohnso@augusta.edu; 4 James and Jean Culver Vision Discovery Institute, Medical College of Georgia at Augusta University, Augusta, GA 30912, USA; 5 VA Augusta Health Care System, Augusta, GA 30904, USA

**Keywords:** SAHA, HDACs, corneal wound healing, diabetes, AQP3

## Abstract

**Highlights:**

**What are the main findings?**

- SAHA improved corneal wound healing in diabetic and normoglycemic mice.
- AQP3 expression was increased at the wound’s edge in the cornea during healing.

**What is the implication of the main finding?**

- SAHA could potentially be used clinically to promote corneal wound healing.
- AQP3 upregulation at the edge of corneal wounds is likely important for healing.

**Abstract:**

Corneal problems, such as delayed and incomplete wound repair, are frequent in diabetes, affecting up to 70% of diabetic patients. In skin, histone deacetylases (HDACs) have been previously found to repress expression of the glycerol channel aquaporin-3 (AQP3), the deficiency of which delays corneal wound healing. We hypothesized that the pan-HDAC inhibitor suberoylanilide hydroxamic acid (SAHA) would improve corneal healing in diabetic mice. Diabetic and normoglycemic C57BL/6J male and female mice were subjected to corneal debridement. Wounds were treated topically with vehicle or SAHA every four hours until they healed. Treatment with SAHA improved wound healing in both normoglycemic and hyperglycemic male mice but, unexpectedly, no changes were detected in female mice. In male mice interleukin-1beta (IL-1β) and tumor necrosis factor (TNF) were significantly increased in diabetic corneas, and SAHA reduced their expression, returning IL-1β and TNF to levels comparable to those in normoglycemic mice regardless of treatment. In normoglycemic male mice, AQP3 levels were not changed in the cornea with SAHA treatment but the expression of AQP3 was increased in the wound’s edge relative to the rest of the cornea. In vitro SAHA treatment of human corneal epithelial cells (HCECs) significantly increased protein expression of AQP3, important for corneal wound healing, but had no effect on ROS production. In conclusion, treatment with SAHA improved corneal wound healing, not only in male mice with diabetes and delayed wound healing but also in normoglycemic male mice; therefore, SAHA could potentially be repurposed as a topical treatment clinically to improve corneal wound healing.

## 1. Introduction

Corneal wounds can occur as a result of injury, trauma, or surgery, and are painful and potentially blinding. Because the cornea is the barrier to the external environment, it is highly susceptible to injury. Normally, the cornea, when injured, heals rapidly to restore its structure and integrity. In some individuals, such as those with diabetes, whose corneas heal slowly or not at all, injuries can cause major visual impairment [1,2,3]. Corneal problems are frequent in diabetes, affecting up to 70% of diabetic patients examined, and range from increased corneal thickness and edema to delayed and/or incomplete wound repair and susceptibility to injury [1,2,3,4].

Aquaporin-3 (AQP3), a water, hydrogen peroxide, and glycerol transporter, has been found to be important in the wound healing process in the skin and cornea, since in both knockout of the gene encoding AQP3 causes a delay in wound healing [5,6,7,8,9]. AQP3 expression is downregulated in multiple tissues in animal models of diabetes [10,11,12,13]. In addition, in humans it has been found that AQP3 is decreased in fetal membranes and in placentas from women with type II diabetes [14,15]. This downregulation of AQP3 in diabetes could be a potential factor, likely of many, for the delayed corneal wound healing observed in diabetic patients.

Histone deacetylases (HDACs), a family of enzymes responsible for the removal of acetyl groups from histone proteins as well as a number of non-histone proteins [16,17,18,19], have been found to regulate the expression of AQP3 in the skin [20,21]. Specifically, HDAC3 modulates cutaneous AQP3 expression such that the inhibition or knockdown of HDAC3 increases AQP3 in the skin and in skin epithelial cells (keratinocytes) [20,21]. In diabetes, it has been found that HDAC expression levels are altered, presenting as a general increase in the expression of several HDACs and correlating with the expression of inflammatory mediators [22,23,24]. In the skin, the selective HDAC6 inhibitor tubastatin A and the selective HDAC3 inhibitor BG-45 have been shown to improve skin wound healing in diabetic mice and to decrease the expression of inflammatory molecules interleukin (Il)-1β, tumor necrosis factor (TNF)-α and Il-6 [25,26]. In rabbit corneas, the use of suberoylanilide hydroxamic acid (SAHA), a pan-specific HDAC inhibitor, reduces corneal haze and decreases the number of myofibroblasts after photorefractive keratectomy [27]. To the best of our knowledge, no studies have examined the use of HDAC inhibitors to enhance corneal wound healing from mechanical injury in diabetes.

We used SAHA, a cancer drug already approved by the Food and Drug Administration (FDA) and marketed as Vorinostat, in vitro using human corneal epithelial cells (HCECs) and in vivo using a streptozotocin (STZ)-induced diabetic mouse model to study corneal wound healing and AQP3 expression. We hypothesized that HDAC inhibition would increase the expression of AQP3 in vitro and in vivo and improve wound healing and reduce inflammatory cytokines in vivo.

## 2. Materials and Methods

A detailed description of the methods is provided in the Supplementary Materials.

### 2.1. Cell Culture

HCECs were obtained from de-identified donor corneal rims, with all cells obtained from these tissues within 24 h of surgery from 3 donors as previously described [28,29]. The donors were a 32-year-old male, a 33-year-old male, and a 58-year-old male. HCECs were cultured in Dulbecco’s Modified Eagle’s medium (with glucose levels of 5.5 mmol/L or 25 mmol/L as indicated) supplemented with FBS (10%), 1% Corning^®^ ITS + Premix Universal Culture Supplement, 25 ng/mL human epidermal growth factor and 40 μg/mL gentamicin on standard tissue culture plates in a humidified incubator at 37 °C with 5% CO_2_, with replacement of culture medium every 2 days. Cells were passaged by detaching them from the culture plate using trypsin (0.025%) + EDTA (0.01%) for 5 min in a humidified incubator at 37 °C.

### 2.2. In Vitro Cell Treatment Plan for SAHA and RGFP966

Detached HCECs were plated at ~75,000 cells per mL in a total volume of 2 mL per well on six-well plates (Catalog No. 07-200-83) in 25.0 mmol/L glucose medium. Once the cells reached 60–70% confluence, they were treated with the pan-specific HDAC inhibitor SAHA (Selleck Chemicals LLC, Houston, TX, USA, dissolved in media at a final DMSO concentration of less than 0.005%) at 1 µM or 2.5 µM or phosphate-buffered saline (PBS) in media as vehicle for 24 h. RGFP996 (Selleck Chemicals LLC, Houston, TX, USA), an HDAC3-selective inhibitor, was dissolved in medium at concentrations of 10 µM or 30 µM and a final DMSO concentration of 0.3%, with DMSO (0.3%) as a control for 24 h. After treatment, cells were harvested (at ~90% confluence) for mRNA analysis or Western blotting.

### 2.3. Cell Plating and Treatment Timeline for Determining the Effect of Medium Glucose Level

HCECs were plated at ~75,000 cells per mL in a total volume of 2 mL per well in six-well plates (Catalog No. 07-200-83) in 5.5 mmol/L glucose medium. After a 24 h recovery period, the medium was replaced with medium containing 25.0 mmol/L glucose or 5.5 mmol/L glucose media osmotically matched with mannitol after the initial osmolarity of both media was measured. After 48 h in the two media, cells were harvested at 85–95% confluency for RT-qPCR or Western blot analysis; cell migration/proliferation or radiolabeled glycerol uptake assays; mitochondrial function using Seahorse Mito-Stress monitoring; or cellular HDAC activity determination. Alternatively, cells were used to measure intracellular ROS as described in the Supplementary Materials.

### 2.4. Cell Migration and Proliferation Assay

Cells were plated on Ibidi Culture-Insert 2 Well in µ-Dish 35 mm (Catalog No. 81176, Ibidi, Fitchburg, WI, USA) at ~100,000 cells/mL and incubated for a 24 h recovery period before treatment as described above using high and normoglycemic conditions with or without SAHA (2.5 µM). Once the cells achieved confluence in both chambers, the barrier was removed to reveal a uniform gap reminiscent of a scratch wound (with no or minimal cell damage compared to scratch wounding). Digital images of the gap closure were taken upon initial removal of the barrier and every 4 h after, until one of the groups attained gap closure. The images were quantified using ImageJ in terms of the area of gap remaining, with the initial wound image set to 100%. Wound healing rates were calculated for each individual group by performing a linear regression analysis. Regression slopes (healing rates) were compared using two-way ANOVA with Tukey’s post hoc tests.

### 2.5. Immunofluorescence Microscopy on Wounded Corneas

All animal procedures were approved by the Institutional Animal Care and Use Committee of Augusta University (protocol #2013-0581 approved 27 March 2025). C57BL/6J mice obtained from Jackson Laboratory (Bar Harbor, ME, USA) were maintained in a temperature- and light-controlled environment with a 12 h day/night cycle with food and water provided ad libitum. After at least a week of acclimation in the animal facility, normoglycemic mice of 20 weeks of age were randomly assigned for treatment with SAHA or PBS. Mice were anesthetized with ketamine and subjected to corneal wounding by scraping the epithelium using an Alger brush on the central 1.5 mm region of the eye; fluorescein, which binds to the exposed stroma but not intact corneal epithelium, was used to visualize the wound. The eyes of the anesthetized mice were treated with PBS every ten minutes until they achieved consciousness to prevent eye desiccation. After wounded mice were provided 1.2 mg/kg of buprenorphine SR as an analgesic, they were treated with either SAHA (10 µM) or PBS vehicle upon wounding and every 4 h until collection of corneas at 8 h and 14 h (n = 5 per group per time point as in [30]). Corneas were collected after euthanasia with isoflurane and cervical dislocation. Upon collection, corneas were fixed in paraformaldehyde (5%) at 4 °C for 75 min, followed by washing with PBS 0.2% Tween-20 at room temperature 3 times for 5 min. Corneas were then placed in 4:1 methanol–DMSO for 2 h, changed to 100% methanol and incubated overnight at 4 °C; they were stored at −20 °C until staining. For staining, corneas were rehydrated through a graded Triton series at 25% Triton in PBS for 15 min, 50% Triton for 15 min and 75% Triton for 10 min. Corneas were then washed 3 times for 30 min at room temperature. Corneas were blocked with 5% BSA blocking buffer for 2 h and then incubated in AQP3 antibody at 1:200 (Alomone Labs, Jerusalem, Israel) overnight at 4 °C with gentle shaking. Cells were washed 7 times for 20 min each in PBS, and then secondary antibody Alexa Fluor™ 594 goat anti-rabbit IgG (1:800) (Invitrogen, Waltham, MA, USA) was applied overnight in the dark at 4 °C with gentle shaking. Corneas were then washed 7 times for 20 min and the edges cut for flat mounting on slides using Fluoroshield™ with DAPI (Invitrogen, Waltham, MA, USA). For imaging, Z-stacks of the corneas were acquired in 16-bit images with a Nikon AX R confocal microscope with NSPARC using 4× Plan-Apochromat (dry)-NA 0.2 lenses (Nikon, Melville, NY, USA) at steps of 13.0 µM. Each cornea was subjected to the same laser intensity while images were acquired. Images for AQP3 fluorescence were then converted to grayscale Z-stack tiff files and analyzed in a blinded manner by Image J 1.53k with the edge of the wound area selected to a depth of ~215 µM from the wound and intensity measured for the whole stack. The whole cornea was then selected and the intensity measured in Image J 1.53k for the whole stack as well. Intensity values were then subtracted from the negative control intensity values before analysis. Images were labeled only by the mouse ear tag number and analyzed in a blinded manner (without the key). Statistical analysis was performed using two-way ANOVA with post hoc Tukey tests in GraphPad Prism 10.0 software.

### 2.6. Corneal Wound Healing In Vivo

A detailed timeline is provided in the Results section. After at least one week of acclimation in the animal facility, ten C57BL/6J male mice acquired from Jackson Laboratory at nine weeks of age were injected with STZ; diabetes was confirmed one week later. After 10 weeks of diabetes, in which the blood glucose levels were determined to be in the range of 249–650 mg/dL (see Supplemental Table S1), these mice and their ten normoglycemic littermates were anesthetized with ketamine and subjected to corneal wounding: the epithelium in the central 1.5 mm region of the eye was scraped using an Alger brush as above. Fluorescein, which binds to the exposed stroma but not intact corneal epithelium, was used to visualize the wound, and anesthetized mice were treated with PBS every ten minutes until mobile to prevent dry eye. After wounded mice were treated with 1.2 mg/kg of buprenorphine SR as an analgesic, images of the fluorescein-visualized wound were taken immediately after wounding and throughout the healing process, until complete healing, using a slit lamp (Topcon, Tokyo, Japan) modified to examine isoflurane-anesthetized mice. The mice were randomly selected to be treated with either SAHA (10 µM) or PBS vehicle upon wounding and every 4 h until the wound healed (n = 4–5 per group). At the time of complete healing, after euthanasia with isoflurane and cervical dislocation, the right and left corneas of the mice were collected for later analysis for mRNA. Corneal images of the healing wound were quantified with ImageJ by measuring the area of the wound at each time point and calculating the percentage of the wound remaining relative to the initial wound area. Images were labeled using the mouse ear tag number and analyzed in a blinded fashion. One normoglycemic mouse died before the wounding experiment was initiated and one diabetic mouse that did not survive until the wound healed was excluded. This experiment was repeated with female mice at 10 weeks of age but the resistance of females to the diabetogenic effects of STZ resulted in delays in inducing hyperglycemia; therefore, most experiments used male mice. All procedures were approved by the Institutional Animal Care and Use Committee of Augusta University (protocol #2013-0581 approved 27 March 2025).

### 2.7. Statistical Analysis

In vitro experiments were performed at least three separate times (n = 3), and values are expressed as mean ± SEM. All data were analyzed with GraphPad Prism 10.0 software using either Student’s T-test, one-Sample T-test, Mann–Whitney, one-way ANOVA with post hoc Tukey tests, Kruskal–Wallis test with Dunn’s multiple comparisons post hoc tests, or two-way ANOVA with post hoc Tukey tests as indicated. Significance was established at *p* < 0.05.

## 3. Results

### 3.1. HDAC Inhibition Increased AQP3 Levels in HCECs Grown in Standard (Hyperglycemic) Conditions

**Figure 1:**
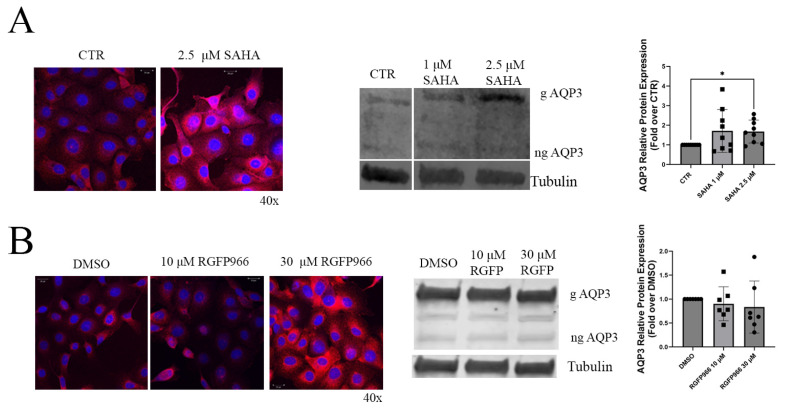
HDAC inhibition in primary human corneal epithelial cells (HCECs) increased AQP3 protein expression. (**A**). Left panel: HCECs were plated on coverslips and once cells reached ~55–60% confluency, they were treated for 24 h in media containing SAHA (2.5 µM) or PBS (CTR). The cells were then fixed with 4% paraformaldehyde and treated with antibodies recognizing AQP3 (red), and nuclei were stained with DAPI (blue). Right panels: At ~60–70% confluency, cells were treated for 24 h in media with PBS (CTR) or SAHA at a concentration of 1 or 2.5 µM. Cells were then lysed after 24 h to collect protein to perform Western blotting (n = 9). (The representative SAHA Western blot image was run on the same gel but in non-adjacent lanes and the intervening lane was removed for the figure.) Western blotting and immunohistochemical staining are representative of at least three separate experiments. Results are shown as means ± SEM, with AQP3 (the sum of the non-glycosylated and glycosylated forms) normalized to tubulin and expressed relative to CTR for analysis by the Kruskal–Wallis test with Dunn’s multiple comparisons test; g = glycosylated and ng = not glycosylated; * *p* < 0.05. (**B**). The HDAC3-selective inhibitor RGFP996 increased AQP3 expression. Left Panel: HCECs were plated on coverslips and once cells reached ~55–60% confluency, they were treated for 24 h in media containing RGFP966 at a concentration of 10 or 30 µM or DMSO at 0.3% (DMSO). The cells were then fixed with 4% paraformaldehyde and treated with antibodies recognizing AQP3 (red), and nuclei were stained with DAPI (blue). Right panels: HCECs at ~60–70% confluency were treated for 24 h in media with the HDAC3-selective inhibitor RGFP966 at a concentration of 10 or 30 µM or DMSO. Cells were then lysed after 24 h to collect protein to perform Western blotting (n = 7). Western blotting and immunohistochemical staining are representative of at least three separate experiments. g = glycosylated and ng = not glycosylated. Results are shown as means ± SEM and were analyzed by a non-parametric Kruskal–Wallis test with Dunn’s multiple comparisons post hoc tests.

Primary human corneal epithelial cells (HCECs) were grown (and treated) in standard medium as in [28] for treatment with SAHA and RGFP966; this standard medium contains 25 mM glucose. Upon reaching 60–70% confluence, cells were treated with or without the pan-HDAC inhibitor SAHA (at 1 and 2.5 µM) for 24 h with harvesting at around 90% confluence. Treatments with SAHA at 1 µM and 2.5 µM each increased protein expression by 1.5-fold, but only the 2.5 µM dose was found to be statistically significant (Figure 1A). AQP3 protein expression was also assessed through immunocytochemistry and found to be increased in HCECs treated with SAHA (Figure 1A). Previous studies in skin and skin cells showed the ability of HDAC3 to bind to the AQP3 promoter and downregulate AQP3 [20,21]. Therefore, the HDAC3-selective inhibitor RGFP996 was also used to examine its effect on the expression of AQP3. However, AQP3 protein levels were not significantly altered by RGFP at 30 µM, as determined by Western analysis, but an increase in expression was observed with immunocytochemistry (Figure 1B).

### 3.2. Hyperglycemic Conditions Altered Inflammatory and Reactive Oxygen Species (ROS) Scavenger Gene Expression but Had Little or No Effect on Mitochondrial Function or ROS Production

**Figure 2:**
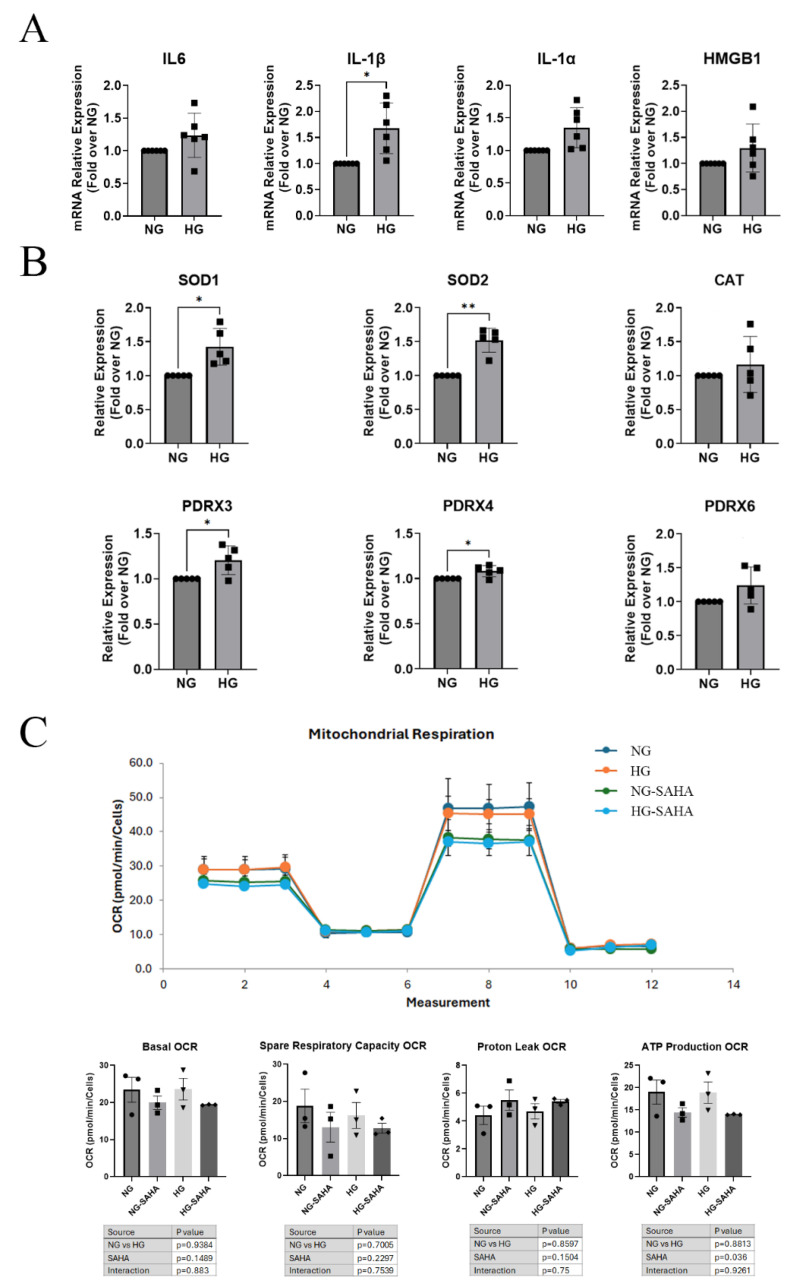
Hyperglycemic conditions increased inflammatory marker and ROS scavenger gene expression in HCECs after a 48 h exposure. (**A**). HCECs grown in hyperglycemic conditions (HG) showed significantly increased IL-1β mRNA compared to cells grown in osmotically matched (mannitol) normoglycemic medium (NG) for 48 h. NG = 5.5 mmol/L glucose; HG = 25 mmol/L glucose. Results are shown as means ± SEM and were analyzed by one-sample T-test (n = 6); * *p* < 0.05. (**B**). HCECs grown in hyperglycemic conditions versus normoglycemic osmotically matched (mannitol) medium for 48 h showed significant increases in mRNA expression for 4 out of 6 ROS scavenger genes. NG = 5.5 mmol/L glucose, HG = 25 mmol/L glucose. Results are shown as means ± SEM and were analyzed by one-sample *T*-test (n = 5); * *p* < 0.05, ** *p* < 0.01. (**C**). HCECs grown in hyperglycemic conditions for 48 h and treated with or without SAHA for 24 h had no change in basal, spare respiratory capacity, or ATP production oxygen consumption rates (OCRs) compared to those grown in normoglycemic osmotically matched (mannitol) medium in the presence or absence of SAHA using Seahorse Mito-Stress assays. NG = 5.5 mmol/L glucose; HG = 25 mmol/L; SAHA = 2.5 µM SAHA. Results are shown as means ± SEM and were analyzed by two-way ANOVA (ANOVA results in tables under graph) with post hoc Tukey tests (n = 3). Note that SAHA induced a slight but significant decrease in ATP production regardless of medium glucose concentration.

Hyperglycemic conditions are known to increase inflammatory mediators and ROS species in multiple tissues [2,31,32,33,34,35]; therefore, hyperglycemic conditions might be expected to increase ROS and inflammatory factors in corneal epithelial cells. To test the effects of hyperglycemic conditions on HCECs, cells were grown for 48 h in hyperglycemic conditions or in normoglycemic medium with osmolarity matched with mannitol. Acute hyperglycemic conditions had no effect on the expression of the damage-associated molecular pattern high-mobility group box 1 (HMGB1) or the inflammatory molecule interleukin (IL)-6; however, the high-glucose medium significantly increased the mRNA levels of the pro-inflammatory mediator IL-1β while IL-1α displayed an increasing trend (Figure 2).

Acute hyperglycemic conditions also increased the mRNA levels of the ROS scavenger genes superoxide dismutase (SOD)-1, SOD2, peroxiredoxin (PRDX)-3 and PRDX4 and tended to increase PRDX6 in HCECs, suggesting possible compensation for an increase in ROS production (Figure 2). There was no effect of high-glucose medium on catalase expression. ROS production in HCECs under acute hyperglycemic conditions was investigated using DCF-DA assays with various concentrations of hydrogen peroxide added as a stressor. Acute hyperglycemic conditions compared to normoglycemic conditions had no effect on ROS production regardless of the hydrogen peroxide concentration; also, SAHA did not alter ROS production (Supplemental Figure S1). To assess whether mitochondrial function was impaired under hyperglycemic conditions, HCECs were grown in hyperglycemic or normoglycemic medium matched osmotically with mannitol for 48 h and treated with or without SAHA for 24 h before initiating Seahorse Mito-Stress assays. Hyperglycemic conditions did not alter any of the mitochondrial functions as assessed by differences in oxygen consumption rate. SAHA treatment, however, did slightly but significantly decrease the production of ATP, without affecting any other mitochondrial functions assessed (Figure 2). 

### 3.3. Acute Hyperglycemic Conditions Did Not Affect HDAC mRNA Levels or Activity, AQP3 Expression, or Migration, but HDAC Inhibition Increased Glycerol Uptake

**Figure 3:**
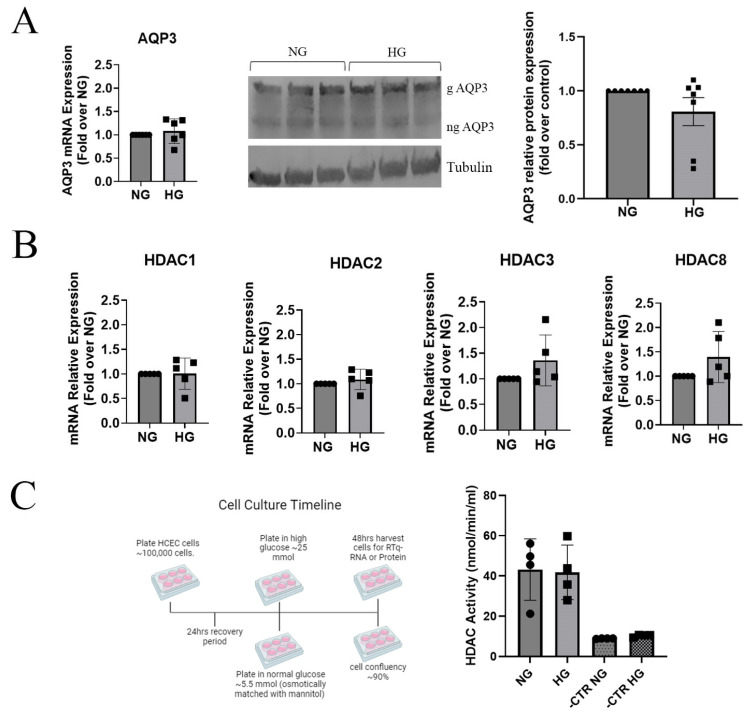
Hyperglycemic conditions for 48 h had no effect on AQP3 expression or HDAC mRNA levels or HDAC activity in HCEC. (**A**) HCECs grown in hyperglycemic (HG) conditions versus normoglycemic (NG) conditions, in osmotically matched media (mannitol), for 48 h showed no significant difference in AQP3 mRNA (n = 6) or protein (n = 7) expression. NG = 5.5 mmol/L glucose; HG = 25 mmol/L glucose. Results are shown as means ± SEM analyzed by one-sample T-test. (**B**). HCECs grown in hyperglycemic conditions versus normoglycemic (NG) conditions in osmotically matched media (mannitol) for 48 h showed a slight increase in HDAC3 and HDAC8 mRNA expression that did not reach statistical significance (n = 5). NG = 5.5 mmol/L glucose; HG = 25 mmol/L glucose. Results are shown as means ± SEM and were analyzed by one-sample T-tests. (**C**). HCECs were grown in black-sided 96-well plates in normal (5.5 mM) (matched osmotically with mannitol) and high (25 mM) glucose conditions for 48 h. HCECs were then incubated with a cell-permeable HDAC substrate for 2 h, and some samples were incubated with pan-HDAC inhibitor trichostatin A as a negative control (-CTR-NG and -CTR-HG). Fluorescence resulting from cleavage of the substrate was then measured as an indicator of HDAC activity. NG = 5.5 mmol/L glucose; HG = 25 mmol/L glucose. Results are shown as means ± SEM analyzed by Student’s T-tests (n = 4).

HDACs have recently been shown to be upregulated in various tissues in diabetes, whereas AQP3 levels are downregulated [22,23,24]; AQP3 has been shown to promote corneal epithelial cell proliferation and migration [9]. We tested the hypothesis that hyper-glycemic conditions for 48 h will decrease AQP3 levels and that HDAC inhibition will improve wound healing in vitro by reversing the decline in AQP3. HCECs were exposed acutely to hyperglycemic conditions for only 48 h because longer culture times in the hyperglycemic environment led to cell death and lower cell numbers compared to the cells in normoglycemic medium. HCECs in hyperglycemic or normoglycemic medium, with osmolarity matched with mannitol, and grown for 48 h showed no significant difference in AQP3 mRNA or protein expression (Figure 3). Hyperglycemic conditions also had no effect on HDAC1, HDAC2, HDAC3, or HDAC8 mRNA expression levels. To verify that the activity of HDAC was also unchanged, an HDAC activity assay was performed in hyperglycemic compared to normoglycemic conditions (again with mannitol matching) for 48 h, and there was no significant effect on total HDAC activity.

**Figure 4:**
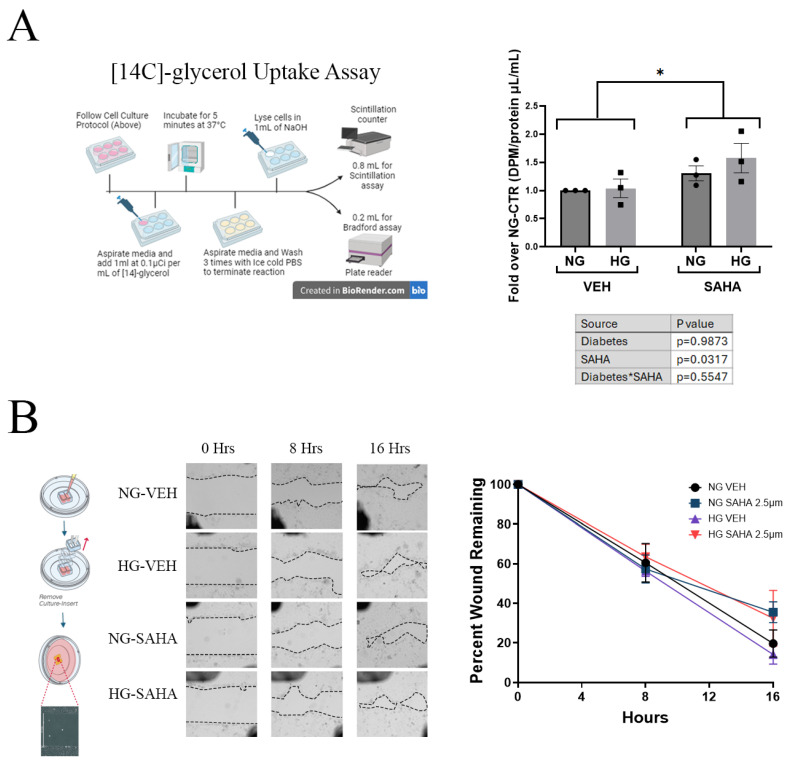
HDAC inhibition increased [^14^C]-glycerol uptake but had no effect on corneal migration/proliferation regardless of glucose concentration. (**A**). After 24 h in hyperglycemic (HG) or normoglycemic conditions, osmotically matched with mannitol (NG), HCECs were treated for an additional 24 h with the pan-specific HDAC inhibitor SAHA at 2.5 µM or vehicle (PBS) in the appropriate HG or NG medium. All groups were then incubated with medium containing 1 µCi/mL [^14^C]-glycerol for 5 min, washed 3 times in cold PBS, and harvested, and the uptake of [^14^C]-glycerol was measured using a scintillation counter. (Please note that HCECs were grown in HG and NG conditions for 48 h total.) NG = 5.5 mmol/L; HG = 25 mmol/L; VEH = PBS; SAHA = SAHA 2.5 µM. Results are shown as means ± SEM and were analyzed with two-way ANOVA with post hoc Tukey tests (n = 3); * *p* < 0.05. (**B**). HCECs grown on Ibidi chambers in HG or osmotically matched (mannitol) NG conditions for 48 h and treated with or without SAHA for 24 h before and during the experiment showed no changes in migration/proliferation. Results are shown as means ± SEM expressed as percent wound remaining analyzed using Image J area (n = 4). NG = 5.5 mmol/L; HG = 25 mmol/L; VEH = PBS; SAHA = SAHA at 2.5 µM and were analyzed by two-way ANOVA (ANOVA results are shown in the table under the graph) with Tukey post hoc tests for percent wound remaining for each individual time point. Asterisk in *SAHA refers to the interaction between SAHA and diabetes and is standard in statistics.

Glycerol uptake, as a measure of AQP3 activity, was assessed in HCECs grown under the above-mentioned glycemic conditions. As shown in Figure 4A, there was no significant difference in [^14^C]-labeled glycerol uptake in HCECs grown in media with a normal or high glucose concentration; however, treatment with 2.5 µM SAHA for 24 h increased glycerol uptake under both conditions, consistent with the ability of this HDAC inhibitor to increase AQP3 levels. A cell migration and proliferation assay was then performed under acute hyperglycemic versus normoglycemic conditions utilizing Ibidi chambers, for which a plug dividing two chambers can be removed to generate a gap with minimal cell injury, allowing cell migration and proliferation to close the gap. Hyperglycemic conditions resulted in no changes in migration or proliferation when compared to normoglycemic conditions. Furthermore, the treatment of cells with the HDAC inhibitor SAHA for 24 h had no significant effect on migration regardless of glycemic condition (Figure 4B).

### 3.4. Wounded Corneas Showed No Difference in Aqp3 Expression Levels by Immunofluorescence of Whole-Mount Corneas, but Aqp3 Levels at the Wound’s Edge Were Increased

**Figure 5:**
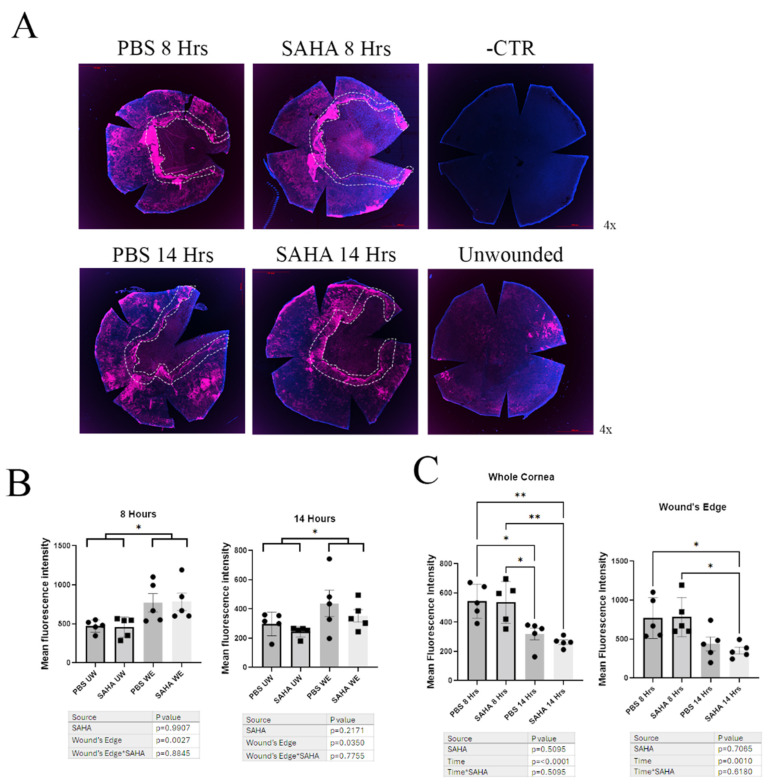
Wounded corneas showed greater AQP3 staining at the wound’s edge in both SAHA- and PBS-treated mice. (**A**). Normoglycemic mice were treated with SAHA (10 µM) or PBS (vehicle control) every 4 h after wounding, and wounded corneas were collected at 8 and 14 h for immunofluorescence staining of AQP3 (red) and DAPI (blue). The wound’s edge is represented in the boxed section of the images. Images are representative and are shown in RGB max intensity from z-stack images. (**B**). AQP3 protein levels are higher at the wound’s edge compared to unwounded sections of the cornea regardless of treatment. Corneas from normoglycemic mice treated with a drop of 10 µM SAHA or PBS (vehicle control) at wounding and every 4 h after were collected at 8 and 14 h after wounding for immunofluorescent staining for AQP3. Z-stack images were analyzed blindly using Image J software in which the wound’s edge (WE) was measured to ~215 µM from the edge and compared to the remaining unwounded (UW) section of the cornea (n = 5). Results are shown as mean fluorescence intensity ± SEM and analyzed by two-way ANOVA with Tukey post hoc tests. * *p* < 0.05. (**C**). AQP3 expression was decreased in wounded corneas as time progressed regardless of treatment. The expression of AQP3 was measured from the same corneas treated with SAHA or PBS every 4 h, as analyzed in panel B. The whole cornea was measured for mean fluorescent values at 8 and 14 h for both SAHA and PBS (left panel). The wound’s edge mean fluorescent values were compared at 8 and 14 h for both SAHA and PBS (right panel) (n = 5). Results are shown as mean fluorescence intensity ± SEM and analyzed by two-way ANOVA with Tukey post hoc tests. * *p* < 0.05, ** *p* < 0.01. Asterisk in *SAHA refers to the interaction between SAHA and diabetes and is standard in statistics.

Since HDAC inhibition with SAHA increased AQP3 levels in vitro, we hypothesized that SAHA would increase Aqp3 levels in mice in vivo. To test this idea, normoglycemic 20-week-old male mice were subjected to corneal wounding with an Alger brush and then treated initially and every 4 h until sacrifice with SAHA or PBS. Aqp3 protein levels were then determined by immunofluorescent microscopy of whole-mount corneas (Figure 5A). Aqp3 levels in the wounded corneas were greater at the wound’s edge at both the 8 h and 14 h time points compared to the remainder of the cornea or to unwounded corneas. This elevation of Aqp3 immunoreactivity was observed regardless of treatment with PBS or SAHA, which had no detectable effect on Aqp3 levels (Figure 5B). Aqp3 levels were greater at the 8 h time point after wounding compared to the later 14 h time point. This increased staining was observed at both the wound’s edge and in the whole cornea, again regardless of treatment (Figure 5C).

### 3.5. Inhibition of HDACs by SAHA Improved Wound Healing in Diabetic and Normoglycemic Male Mice but Not Female Mice

**Figure 6:**
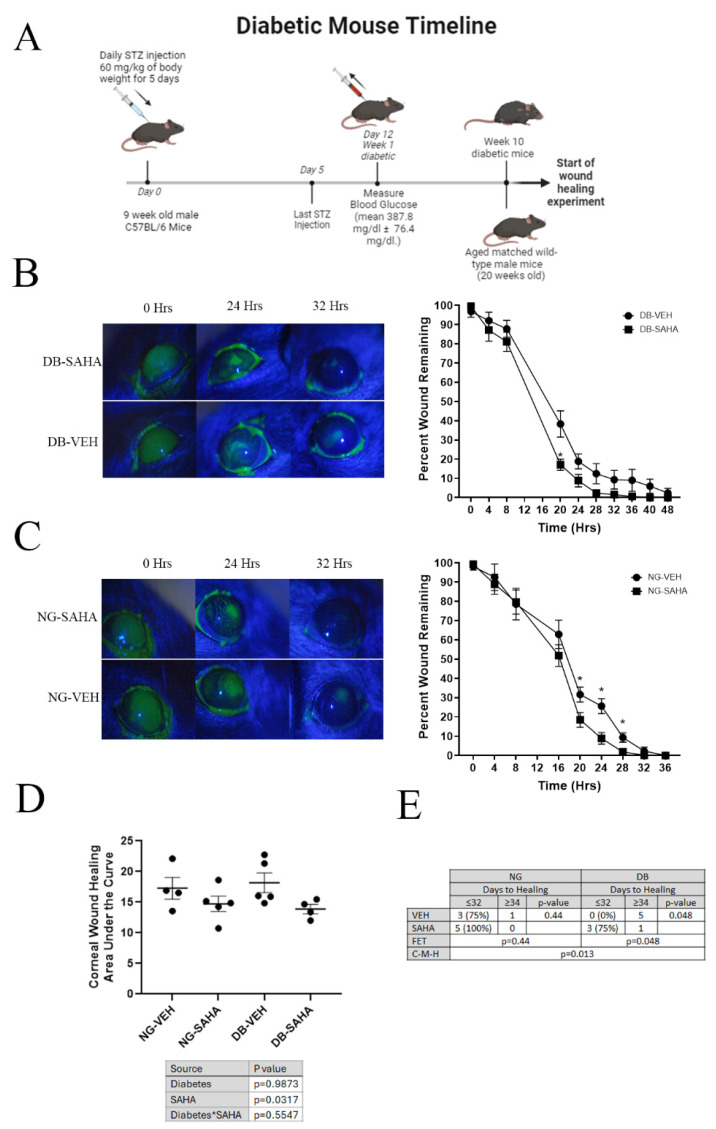
SAHA accelerated corneal wound healing in C57BL/6 male mice. (**A**). Timeline for induction of diabetes in mice and wound healing schematic. (**B**). SAHA improved wound healing in diabetic male mice. Diabetic (DB) C57BL/6 male mice treated with a drop of 10 µM SAHA or PBS (vehicle control) every 4 h after wounding were anesthetized with isoflurane and their wounds visualized with fluorescein to monitor healing. Results are shown as means ± SEM and were analyzed by Student’s *t*-test at each time point (n = 4–5); * *p* < 0.05; DB = diabetic; VEH = vehicle; SAHA = SAHA (10 µM). (**C**). SAHA accelerated corneal wound healing in normoglycemic C57BL/6 male mice. Mice treated with a drop of 10 µM SAHA or PBS (vehicle control) every 4 h after wounding were anesthetized with isoflurane and their wounds visualized with fluorescein to monitor wound healing. Results are shown as means ± SEM and were analyzed by Student’s *t*-test at each time point (n = 4–5); * *p* < 0.05. NG = normoglycemic; VEH = vehicle; SAHA = SAHA (10 µM). (**D**). SAHA improved wound healing regardless of mouse glycemic status. The area under the curve for normoglycemic and diabetic mice treated with or without SAHA was analyzed by two-way ANOVA (results are shown in the table under the graph) and demonstrated that treatment with SAHA improved corneal wound healing compared to the vehicle (n = 4–5). NG = Normoglycemic; DB = diabetic; VEH = vehicle; SAHA = SAHA (10 µM). (**E**). Male diabetic mice healed more slowly than normoglycemic mice. We examined whether the mice healed by or at 32 h or not by performing within-disease Fisher’s exact tests (FET) and found that there is not a significant association between SAHA treatment and healing in normoglycemic mice, but healing in SAHA-treated diabetic mice was significantly more rapid than in diabetic mice treated with PBS. Therefore, a Cochran–Mantel–Haenszel test (C-M-H) was performed and resulted in a significant difference in the general association between normoglycemic and diabetic mice with SAHA treatment. Asterisk in *SAHA refers to the interaction between SAHA and diabetes and is standard in statistics.

Although these results suggested that our initial hypothesis that HDAC inhibition with SAHA would increase Aqp3 levels in vivo was incorrect, we were nevertheless interested in the possibility that SAHA might still improve corneal wound healing. This idea was based on the fact that data in the literature suggest anti-inflammatory properties of HDAC inhibition (reviewed in [36,37]), and excessive or chronic inflammation delays corneal wound healing (reviewed in [38]). Male mice were administered STZ injections at 9 weeks of age, and at 10 weeks of age they were confirmed to be diabetic with an average blood glucose level of 377.1 ± 80 mg/dL (Supplemental Table S1). After 10 weeks of hyperglycemia, the diabetic male mice and a normoglycemic cohort were subjected to wounding using an Alger brush. Measurement of the area of the wound remaining over time showed that SAHA treatment accelerated wound healing in the diabetic male mice compared to the vehicle control. The wound area remaining was 30% less in the SAHA-treated mice at 20 h, and this trend of improved wound healing in the SAHA-treated diabetic mice was maintained until the wounds healed (Figure 6). Normoglycemic male mice also demonstrated a significant improvement in wound healing with SAHA treatment, such that at 20 h there was less wound remaining in the SAHA-treated mice, and this trend also continued until the wounds healed. Comparing the areas under the curve, for which a lower area indicates faster healing, treatment with SAHA accelerated wound healing in the mice regardless of glycemic status, with diabetes showing no significant effect on the rate of healing by two-way ANOVA. Nevertheless, it was noted that there appeared to be a delay in the complete closure of the wound at later time points in diabetic compared to normoglycemic male mice. Indeed, in the normoglycemic mice treated with PBS, three of four (75%) were healed by or at 32 h, whereas in the PBS-treated diabetic animals, zero of five were healed (0%), suggesting the tendency of diabetes to delay wound healing. Using another statistical analysis in which a Cochran–Mantel–Haenszel test was applied after wound healing was dichotomized by whether or not the corneas were healed by or at 32 h, SAHA was found to significantly promote healing in diabetic (*p* = 0.048) but not normoglycemic (*p* = 0.44) male mice. Therefore, there was a significant difference in general association between normoglycemic and diabetic mice with SAHA treatment (*p* = 0.013).

The wound healing experiment was repeated using female mice. Female mice exhibited no difference in corneal wound healing with SAHA treatment or glycemic status. However, a comparison of the female and male mice showed that wound healing was significantly slower in female mice. Thus, the female mice healed on average at approximately 4 days while the male corneas healed at around an average of 1.65 days (Supplemental Figure S2).

### 3.6. Healed Corneas Showed Significant Differences in Inflammatory Marker Expression in Vehicle-Treated Compared to SAHA-Treated Diabetic Male Mice

**Figure 7:**
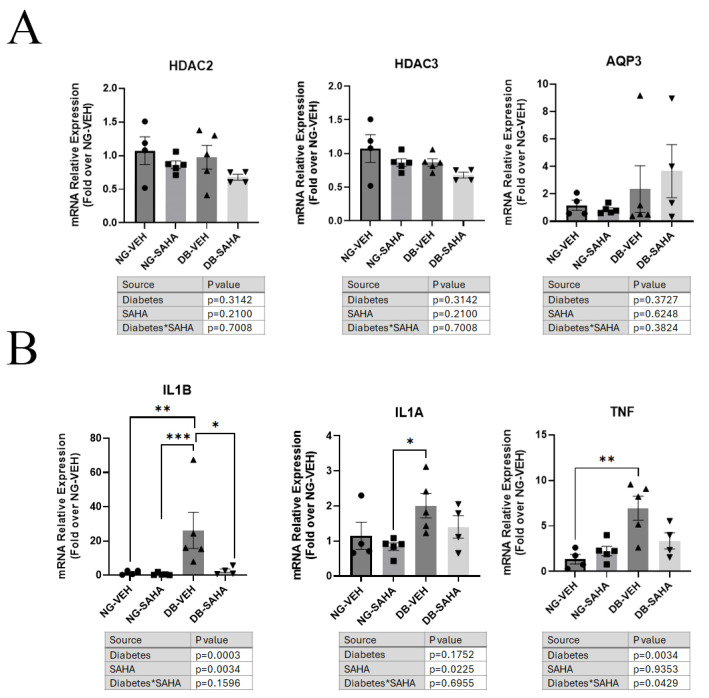
Expression of inflammatory molecules interleukin-1β (IL1B) and interleukin-1α (IL1A) was reduced in healed diabetic male mice treated with SAHA, but AQP3 and HDAC expression was no different in healed corneas from normoglycemic or diabetic mice treated with SAHA or not. (**A**). Healed corneas collected from diabetic and normoglycemic mice treated with or without SAHA were collected and digested with Trizol for RNA extraction for RT-qPCR. Analysis was performed by two-way ANOVA (results are shown in the table under the graph) and post hoc Tukey tests on delta-Ct values (n = 4–5). NG = normoglycemic; DB = diabetic; VEH = vehicle; SAHA = SAHA (10 µM). (**B**). Healed corneas collected from diabetic and normoglycemic mice treated with or without SAHA were collected and digested with Trizol for RNA extraction for RT-qPCR and analyzed using the delta-delta Ct method. Results are shown as means ± SEM. Analysis was performed by two-way ANOVA (results are shown in the tables under the graphs) and post hoc Tukey tests on delta-Ct values (n = 4–5). * *p* < 0.05, ** *p* < 0.01, *** *p* < 0.001; NG = normoglycemic; DB = diabetic; VEH = vehicle; SAHA = SAHA (10 µM). Asterisk in *SAHA refers to the interaction between SAHA and diabetes and is standard in statistics.

Injured corneas collected at healing were processed for mRNA isolation to determine the expression of Hdacs, Aqp3, ROS-detoxifying genes and inflammatory mediators by RT-qPCR. Hdac2, Hdac3, and Aqp3 mRNA levels were no different in normoglycemic versus diabetic mice regardless of treatment (Figure 7A). Conversely, TNF mRNA levels were higher in diabetes, with a significant difference between normoglycemic and diabetic mice treated with vehicle. There was a significant interaction by two-way ANOVA, with SAHA tending to decrease TNF in the diabetic mice and raise the cytokine’s expression in the normoglycemic cohort (Figure 7B). On the other hand, SAHA significantly decreased Il-1α mRNA levels under both conditions, with Il-1α expression significantly greater in the diabetic mice treated with vehicle compared to the normoglycemic mice treated with SAHA. Il-1β, however, was 20-fold higher in the healed corneas of diabetic mice compared to normoglycemic animals. Treatment of diabetic mice with SAHA returned the expression of Il-1β to values similar to those in the normoglycemic vehicle controls, indicating the capacity of SAHA to reduce the expression of certain pro-inflammatory mediators (Figure 7B). There was no significant difference in the expression of the ROS scavenger genes Sod1 or Sod2 between normoglycemic or diabetic mice regardless of treatment (Supplemental Figure S3A). Likewise, there was also no difference in expression of Hmgb1 or the inflammatory molecule Il6 in the healed corneas with or without SAHA treatment (Supplemental Figure S3B).

## 4. Discussion

By the year 2040, it is predicted that 10.4% of all adults worldwide aged 20–79 will have diabetes [39], and there are currently over 34 million Americans with this disease [2,39]. Corneal problems are frequent in diabetes, affecting up to 70% of examined diabetic patients, and range from increased corneal thickness and edema to delayed and incomplete wound repair and susceptibility to injury, all of which can potentially lead to vision loss [1,2,3,4]. Any treatment to help improve corneal wound healing in people with diabetes would be very beneficial.

Wound healing is a multistep process coordinated by many cell types, including immune cells [38,40]. Using an in vitro scratch closure assay, it has been found that hyperglycemia induces a delay in wound healing in confluent HCECs [41]. When we examined wound closure in HCECs using Ibidi separation chambers to form a uniform wound with minimal cell injury, no difference was observed in the rate of HCEC scratch wound closure, regardless of glycemic condition, or in the presence or absence of SAHA. However, we detected an improvement in the rate of wound healing in vivo in diabetic and normoglycemic mice treated with SAHA. The explanation for this disparity may be that SAHA’s main target in the wound healing process could be a cell type other than corneal epithelial cells, such as immune cells, or one activated through a process related to the scratching/debridement process itself. Thus, it can be speculated that mechanically scratching a confluent monolayer of cells for the wound closure assay in vitro or debriding the cornea in vivo results in the release of damage-associated molecular patterns (DAMPs) that can activate pattern recognition receptors such as toll-like receptors, thereby promoting pro-inflammatory signaling [38]. SAHA may inhibit this inflammatory process, as we observed in terms of the SAHA-induced reduction in Il1b in healed diabetic mouse corneas.

It has been found in skin cells and the skin that the selective HDAC6 inhibitor tubastatin A helps improve wound healing in vitro under high-glucose conditions and in vivo in a diabetic rodent model [25]. Tubastatin A and Class I HDAC inhibitor BG-45 were also found to reduce inflammatory molecules, mainly IL-1β, IL-6 and TNF-α, produced by macrophages [25,26]. Interestingly, in the current study, IL-1β was the inflammatory marker that was significantly increased under hyperglycemic conditions in HCECs in vitro and in the healed corneas of diabetic mice treated with vehicle. TNF and IL-1β are produced mainly by macrophages during wound healing and help drive the injury and subsequent resolution phase of the wound healing response [40]. Chronic wounds in humans and wounds with impaired healing in diabetic mice show sustained inflammasome activation in macrophages and elevated levels of IL-1β and TNF-α [42,43,44,45]. Indeed, disruption of IL-1β generation using inflammasome inhibitors or inhibition of its action using an IL-1β-specific antibody reduces inflammation and accelerates wound healing [46]; however, complete depletion of macrophages impairs wound healing [47]. In the cornea, it has been found that IL-1β and TNF-α levels are increased during wounding, peaking at 12 h; however, diabetic mice show a sustained increase in IL-1β and TNF-α levels that causes a delay in wound healing [45]. In HCECs under hyperglycemic conditions, there was a 1.5-fold increase in IL-1β expression, suggesting that the increase in IL-1β levels observed in diabetes could be at least partially due to hyperglycemia. In our study, IL-1β expression was markedly higher in diabetic mice (by ~24-fold), and treatment with SAHA resulted in lower expression (20-fold less), suggesting that the reduction in IL-1β levels may be an important factor in improving corneal wound healing. Furthermore, our study found that TNF expression levels were reduced in diabetic mice treated with SAHA. It has been previously found that TNF-α levels and HDAC expression and activity are increased in diabetic patients with delayed wound healing [22,24,48].

In skin cells, AQP3 expression is regulated, at least in part, by HDACs, and more specifically by HDAC3, such that the inhibition of HDACs by SAHA or HDAC3 selectively by RGFP966, was found to increase AQP3 expression [20]. In the current study, we observed an increase in AQP3 levels upon exposure of HCECs to RGFP966 by immunofluorescence (with no significant increase observed with Western analysis), suggesting a possible similarity to skin epithelium. Further, in patients with diabetic foot ulcers it was found that HDAC1, 3, 4 and 11 were upregulated [24]. In particular, HDAC3 has been found to play a role in inflammatory molecule upregulation while HDAC1 and 2 are not key factors [48]. In fact, in patients with type II diabetes, HDAC3 appears to be upregulated compared to other HDACs [22]. Together, these data suggest that the selective HDAC3 inhibitor RGFP996 might enhance corneal wound healing to a greater extent and with fewer side effects; however, further research is needed to test this idea.

SAHA was ineffective in improving corneal wound healing in female mice, regardless of their glycemic status. In fact, no differences were observed in wound healing in female mice, regardless of blood glucose status or treatment with SAHA. It is well known that female mice have a resistance to diabetes induced by STZ, displaying a lesser diabetic phenotype than male mice [49], which may explain, in part, why no differences were observed in the diabetic compared to normoglycemic female mice. However, even the male diabetic mice in our study did not display as marked a delay in wound healing compared to their normoglycemic counterparts as has previously been reported ([50,51]); this result could be due to the mice being hyperglycemic for only 10 weeks, which may not be sufficient to induce a striking delay in the wound healing phenotype and is a potential limitation of the study. Nevertheless, it was noted that there appeared to be a delay in the complete closure of the wound at later time points in diabetic compared to normoglycemic male mice. Indeed, in the normoglycemic male mice treated with PBS, three of four (75%) were healed by or at 32 h, whereas in the PBS-treated diabetic animals zero of five were healed (0%), suggesting the tendency of diabetes to delay wound healing.

Previous research on whether sex is a biological factor in corneal wound healing is mixed, with some studies showing that female mice exhibit slower corneal wound closure than male mice [52,53], whereas other studies in mice and in rabbits reported no sex differences in corneal wound healing [54,55]. In our study, male mice healed on average approximately 2 days faster than females. Female mice may heal more slowly due to estradiol downregulating cornea-expressed 15-lipoxygenase (15-LOX) and receptors for lipoxinA4 (LXA4), both of which have been implicated in an intrinsic lipid circuit that regulates corneal inflammation and wound healing [53]. Differences in the estrous cycle could explain the extreme variance that we observed in healing rate in the female mice, although over the course of the average 4-day healing time, all female mice would have likely experienced all stages. Unfortunately, estrous cycles were not determined for the female mice, so further research is needed to test this idea. Alternatively, it has been found that female mice respond to stress with greater increases in the levels of corticosterone (the primary mouse glucocorticoid) than males [56]. Since glucocorticoids are known to delay wound healing [57,58], this sex difference may have played a role in the observed delay in wound healing, with SAHA unable to overcome the steroid’s inhibitory action. Further research is needed to confirm the differences in corneal wound healing in female versus male mice under various conditions (e.g., stress, ovariectomy), as well as in human cornea epithelial cells from female donors, and to determine the mechanism underlying these disparities.

An accumulation of ROS can cause mitochondrial injury, as seen in corneal endothelial cells [34,35,59]. ROS have also been linked to the etiology of corneal surface disorder in blink-suppressed dry eye disease [33]. Hyperglycemic conditions have been shown to increase ROS production in HCECs within 24 h [3]. However, our results differed from those of Xu et al. [3], as there was no difference detected in ROS production at 48 h, although there was an increase in the expression of inflammatory mediators and ROS scavenger genes, suggesting the possibility that there was an increase in ROS production for which the cells were able to compensate. Based on this idea, mitochondrial function in HCECs was tested, as well as the effect of SAHA on mitochondrial function. Exposure of HCECs to acute hyperglycemic conditions had no effect on mitochondrial function in terms of basal respiration, spare respiratory capacity, proton leak, or ATP production, as monitored with oxygen consumption rate using Seahorse Mito-Stress assays. The lack of effect may again be due to a 48 h exposure to hyperglycemia being insufficient to induce changes in mitochondrial function relative to diabetes, which is a chronic disease. In a human telomerase-immortalized corneal epithelial (hTCEpi) cell line, it was found that mitochondrial function was unaffected by hyperglycemia until 5 days had passed, at which time hyperglycemia impaired mitochondrial function [60]. Interestingly, the short-term use of SAHA for 24 h slightly, but significantly, reduced ATP production but did not change any other parameters of mitochondrial function, suggesting that short-term exposure to SAHA has little or no harmful effect on HCECs. Indeed, it has been found that in corneal limbal cells SAHA at concentrations of 10 µM does not alter viability, and apoptosis is not increased [61]. In addition, in a photorefractive keratectomy-induced rabbit model of corneal haze, SAHA administration after injury decreases corneal haze with no effect on keratocyte density or apoptosis, nor any compromise of the corneal endothelial phenotype four months after treatment [27]. In another study, the use of SAHA topically for 7 days on mouse corneal epithelium had no effect on corneal epithelial cell apoptosis, suggesting the likely safety of SAHA for potential use to improve acute wound healing [62].

AQP3 is an important protein involved in corneal wound healing and is downregulated in multiple tissues in diabetes [5,6,7,8,9,10,11,12,13,14,15]. A potentially important function of AQP3 is its colocalization with phospholipase D2 (PLD2) in both the skin and cornea [63,64]. In skin epithelial cells (keratinocytes), this colocalization is thought to allow the glycerol transported by AQP3 to be converted by PLD2 to phosphatidylglycerol (PG), which serves as a lipid signal [65,66]. The specific PG, dioleoylphosphatidylglycerol (DOPG), has been shown to improve delayed corneal wound healing in AQP3 knockout mice, as well as in wild-type mice [63], suggesting the importance of the AQP3/PLD2/PG pathway in corneal wound healing. In addition, in the skin, AQP3 is involved in the hydration of this organ, predominantly through its transport of glycerol rather than water, as suggested by studies in AQP3 knockout mice [7]. AQP1 and AQP5, and likely AQP3, play an important role in maintaining normal corneal hydration by acting as passive transport pathways for water out of the cornea after the creation of an osmotic gradient by the endothelial cells [67]. The hydration of the cornea affects its thickness, such that greater hydration increases thickness which then lowers tensile strength, making the cornea more prone to wounding [68,69]. Excessive hydration (i.e., corneal edema) can also lead to reduced visual acuity [70].

In our in vitro study, the pan-specific HDAC inhibitor SAHA increased AQP3 mRNA and protein expression and enhanced glycerol uptake, a measure of AQP3 function, in HCECs [71,72]. In additional in vitro studies, short-term (48 h) hyperglycemic conditions did not alter AQP3 or HDAC expression or activity, although as mentioned in the Introduction, diabetes decreases AQP3 and increases HDAC expression in vivo [10,11,12,13]. This lack of change with short exposures versus with diabetes could be due to the fact that diabetes is a multifaceted chronic disease that takes time to develop, such that acute hyperglycemia was not sufficient to mimic the effects of diabetes. Other factors altered by long-term hyperglycemia might also be involved. For example, in HaCaT cells, a cell line derived from human skin epithelial cells, incubation under hyperglycemic conditions did not affect AQP3 expression; however, treatment with TNF-α, the corneal expression of which we found to be increased with diabetes in mice in vivo, decreased AQP3 levels [10].

In our in vivo study, wounded mouse corneas were found to exhibit an increase in Aqp3 levels at 8 h after wounding; this increase was diminished, although still elevated relative to unwounded corneas, at 14 h. An increase in Aqp3 immunoreactivity in the region near the wound’s edge compared to the remainder of the cornea was observed, suggesting the likely importance of Aqp3 for corneal wound healing. It should be noted that AQP3 expression is increased in and/or near the wound’s edge in multiple skin wound types, including abrasion wounds, frost erythema, and burn [73,74]; however, to our knowledge, ours is the first report showing an increase in Aqp3 levels at the edge of cornea wounds, which declines with healing. However, the use of SAHA had no effect on Aqp3 levels at either 8 h or 14 h after wounding in the normoglycemic mice either in the whole cornea or at the wound’s edge. A potential limitation of this experiment is that immunofluorescence may not be ideal for determining small changes in protein levels. However, with increases in the quality of fluorescent probes and confocal microscopes, immunofluorescence has become a semi-quantitative method [75] to measure protein levels, especially when the number of cells of interest in a tissue are limited, and proper precautions are taken [75,76]. Since much of the epithelium was removed with the wounding protocol, this method was selected due to concerns about obtaining sufficient protein for Western blotting. Although AQP3 expression is regulated partly through HDACs in the skin [20], the regulation of AQP3 in corneal wounding appears to be different. After HDAC inhibition in vitro by SAHA in HCECs, there was a small but significant increase in AQP3 expression and glycerol uptake. However, in the wounded corneas of mice treated with SAHA there were no changes in AQP3, thereby refuting our initial hypothesis that HDAC inhibition would accelerate corneal wound healing by stimulating AQP3 expression in the cornea. Despite no changes in AQP3 expression from the use of SAHA, HDAC inhibition did improve wound healing in both normoglycemic and diabetic mice, probably through the downregulation of inflammation/immune system activation. We are currently performing RNA-sequencing analysis of differentially expressed genes in wounded corneas treated with SAHA versus vehicle control to determine other potential pathways underlying the ability of SAHA to improve corneal wound healing.

## 5. Conclusions

We found that topical application of the pan-HDAC inhibitor SAHA improved corneal wound healing in male diabetic mice as well as in their normoglycemic counterparts. Furthermore, SAHA treatment reduced inflammatory cytokines, most notably IL-1β and TNF, which are known to help drive the injury phase of wound healing; however, when dysregulated, these can impair wound healing [40,42,43,44]. Although SAHA did not increase Aqp3 expression in wounded corneas in our experiments, we observed elevated Aqp3 immunoreactivity specifically at the wound’s edge, suggesting the likely involvement of AQP3 in wound healing. In vitro, HCECs treated with SAHA showed a notable increase in the expression and function of AQP3, an important protein for wound healing in the skin and cornea [5,6,7,8,9]. Furthermore, short-term exposure to SAHA did not appear to increase ROS production or markedly alter mitochondrial function in HCECs. In conclusion, our results support the idea that the Food and Drug Administration-approved drug SAHA (vorinostat) could potentially be repurposed for topical application to improve corneal wound healing; however, further research is necessary.

## Data Availability

The raw data supporting the conclusions of this article will be made available by the authors on request.

## Supplementary Materials

The following supporting information can be downloaded at https://www.mdpi.com/article/10.3390/cells14231880/s1: Detailed and additional methods; Figure S1: Neither glucose status nor SAHA treatment altered ROS levels with or without exposure to hydrogen peroxide as a stressor; Figure S2: Female mice healed more slowly than male mice and showed no difference in wound healing regardless of glycemic status and/or treatment with SAHA; Figure S3: ROS Scavenger genes Sod1 and Sod2, damage-associated molecular pattern Hmgb1, and inflammatory molecule Il6, exhibited no change in expression regardless of diabetes or SAHA treatment; Table S1: Blood glucose levels for diabetic male mice over time.

## Abbreviations

The following abbreviations are used in this manuscript:

ANOVA	Analysis of variance
AQP3	Aquaporin-3
DMSO	Dimethylsulfoxide
FDA	Food and Drug Administration
HCEC	Human corneal epithelial cells
HDAC	Histone deacylatase
IL-1β	Interleukin-1beta
Il-1α	Interleukin-1alpha
PBS	Phosphate-buffered saline
PRDX	Peroxiredoxin
ROS	Reactive oxygen species
SAHA	Suberoylanilide hydroxamic acid
SEM	Standard error of the mean
SOD	Superoxide dismutase
STZ	Streptozotocin
TNF	Tumor necrosis factor
