# Supplementary material for: Histone Deacetylase Inhibition Enhances AQP3 Levels in Human Corneal Epithelial Cells and Corneal Wound Healing in Normoglycemic and Diabetic Male Mice
Source: Cells. 2025 Nov 27;14(23):1880. doi: 10.3390/cells14231880 (PMC12691540; doi:10.3390/cells14231880)
Supplement: Supplementary file 1 [file cells-14-01880-s001.zip › cells-3936327-supplementary.pdf]

Supplemental Materials

Detailed and Additional Methods:

Cell Plating and Treatment Timeline for Determining the Effects of Media Glucose Level: HCECs were plated at ~75,000 cells per mL in a total volume of 2 mL per well on six-well plates (Catalog No. 07-200-83) in 5.5 mmol/L glucose media. After a 24-hour recovery period, the medium was replaced with medium containing 25.0 mmol/L glucose or 5.5 mmol/L glucose media osmotically matched with mannitol. Initial osmolarity of both media types was measured, and mannitol solution was added to match the osmolarity. After 48 hours in the two media, cells were harvested at 85-95% confluency for RT-qPCR or Western blot analysis; cell migration/proliferation or radiolabeled glycerol uptake assays; mitochondrial function using Seahorse Mito-Stress measurements; or cellular HDAC activity determination. Alternatively, cells were used to measure intracellular ROS as described below.

Western Blotting: Cells were harvested after their respective treatments at ~90-95% confluence using hot lysis buffer [3% SDS, 0.1875M Tris-HCl (pH 8.5), and 1.5mM EDTA] for 5 minutes followed by scraping. After repeated pipetting of the sample for homogenization, 20 µL of sample was set aside for determination of protein content using the Bradford assay with bovine serum albumin as the standard. The samples were then mixed with 3x sample buffer (30% glycerol, 15% beta-mercaptoethanol, 1% bromophenol blue, 54% water) and boiled for 5 minutes. Equal amounts of sample protein were loaded onto SDS-PAGE 15% precast gels (Bio-Rad) and, following separation by electrophoresis, transferred to PVDF membrane using a Trans-Blot Turbo Transfer system (Bio-Rad). Membranes containing transferred proteins were blocked with Intercept® (PBS) Blocking Buffer for approximately 1 hour and then incubated with gentle shaking overnight at 4°C with anti-rabbit polyclonal AQP3 antibodies (Alomone

Labs, Jerusalem, Israel or Novus, Centennial, CO), followed by washing and incubation with fluorescent secondary antibodies conjugated to infrared IRDye® 800CW dye for 1 hour at room temperature in the dark. Tubulin was used as a loading control and was visualized with a mouse monoclonal antibody and the appropriate secondary antibody conjugated to IRDye® 680RD and measured at 700nm. Blots were imaged using a LiCor Odyssey® FC Imager and quantified using Licor software. Results are presented as the fold over control within each experiment, with values representing the means ± SEM of at least 5 separate experiments.

RT-qPCR: Following treatment as described above, total RNA was extracted and purified following the manufacturer's protocol for PureLink™ RNA Mini kits (Thermo Fisher Scientific). The quality and quantity of isolated RNA was assessed using a Nanodrop instrument. Reverse transcription was performed using High-Capacity cDNA Reverse Transcription kits (Thermo Fisher Scientific) according to the manufacturer's protocol. The cDNA was diluted at a 1:5 ratio with DNase-free water. Equal quantities of diluted cDNA were used in qPCR reactions, and gene expression was monitored using Taqman probes for all genes measured. The quantitative PCR reaction was performed using Fast Reagent PCR Master Mix (Thermo Fisher Scientific) and the StepOnePlus Real-Time PCR System (Thermo Fisher Scientific) as per the manufacturer's protocol. Relative gene expression was analyzed by the delta-delta C_t method using the average of RPLP0 and GAPDH as the endogenous housekeeping genes, normalized to the respective control group and visualized as $2^{-\Delta\Delta Ct}$ values.

Immunocytochemistry: HCECs were plated on coverslips at ~37,500 cells/mL in 25.0 mmol/L glucose medium; once cells reached ~55-60% confluency, they were incubated for 24 hours in medium containing RGFP966 at a concentration of 10 or 30 μM, SAHA at 2.5 μM or vehicle [PBS for SAHA and 0.3% DMSO

at RGFP966 (control)]. The cells were then fixed with 4% paraformaldehyde for 10 minutes, permeabilized with 0.1% Triton X-100, and blocked with 5% BSA and normal goat serum for 1 hour. Cells were incubated overnight with primary antibodies recognizing AQP3, and then with fluorescent dye-conjugated secondary antibodies for 1 hour in the dark. Fixed cells were mounted with ProLong Gold Anti-Fade reagent containing 4′,6-diamidino-2-phenylindole (DAPI) and visualized with a confocal microscope (Zeiss, Jena, Germany).

[^{14}C]-Glycerol Uptake: After HCECs were grown and treated in high (25 mM) or normal (5.5 mM) glucose isosmotically matched medium with or without SAHA, the medium was changed and the cells were incubated for exactly 5 minutes in their respective conditions with the addition of 20 mM HEPES and 0.1μCi/mL [^{14}C]-glycerol. The cells were then rapidly washed 3 times in cold PBS lacking divalent cations and solubilized in 1 mL of 0.3M NaOH. Aliquots of solubilized cells (0.8 mL) were subjected to liquid scintillation counting and protein estimation. Data are expressed as fold over control (with normal glucose or NG set as 1) for each experiment, with DPM normalized using protein concentration determined using Bradford assays.

HDAC Activity: HDAC activity was assessed using Cayman's HDAC Cell-Based Assay Kit (Ann Arbor, MI) following the manufacturer's protocol. Briefly, cells were grown in black-sided 96-well plates in high (25 mM) and normal (5.5 mM with mannitol to match osmolarity) glycemic conditions for 48 hours. Cells were then incubated with a cell-permeable HDAC substrate for 2 hours in fresh media in a CO_2 incubator, with the pan-HDAC inhibitor trichostatin A added to some wells to serve as a negative control. Cells were then lysed in lysis/developer buffer and incubated for 15 minutes. Fluorescence was measured using a Synergy HT microplate reader from Bio-Tek Instruments (Winooski, VT, USA) with Gen5 analysis

software, using an excitation wavelength of 340-360 nm and an emission wavelength of 440-460 nm. A standard curve was conducted on the same plate, as recommended by the protocol. HDAC activity was measured by correcting the fluorescence reads with the blank measurement, and HDAC activity was calculated for each well using the equation: HDAC Activity (nmol/min/ml) = Corrected Fluorescence Value - (y-intercept of standard curve) / (Slope of standard curve x Incubation Time [120 minutes]), as shown below. Statistical analysis was performed using two-way ANOVA and Tukey post-hoc tests (n = 4).

$$\text{HDAC Activity}\left(\frac{\frac{\text{nmol}}{\text{min}}}{\text{mL}}\right) = \frac{\text{Corrected Fluorescence Value} - (\text{y} - \text{intercept of standard curve})}{\text{Slope of Standard Curve x Incubation Time [120 minutes])}}$$

HCEC ROS Assay: HCECs were plated on black 96-well plates at ~75,000 cells/mL. The treatment conditions and timeline followed the plan described in the section on Cell Plating and Treatment Timeline. ROS were measured using the OxiSelect™ Intracellular ROS Assay Kit (Cell Biolabs, Inc., San Diego, CA), following the manufacturer's protocol. Briefly, cells were washed 3 times with PBS and incubated with medium without phenol red containing DCFH-DA (0.5 mM) for 40 minutes. The cells were then washed 3 times with PBS and treated with phenol red-free medium in their respective glucose conditions of 25.0 mM or 5.5 mM (matched isosmotically with mannitol) with the addition of either 0, 10, 100 or 1000 mM hydrogen peroxide. Fluorescence measurements were recorded on a plate reader at excitation and emission wavelengths of 480 nm and 530 nm, respectively, at the start of hydrogen peroxide treatment and every 15 minutes for 1 hour. This experiment was conducted three separate times and the data analyzed using two-way ANOVA and Tukey post-hoc tests at each time point.

Mitochondrial Function Determination using Seahorse Mito-Stress Assays: Cells were cultured as described above but were plated on a Seahorse assay culture plate at ~75,000 cells/mL. Seahorse assays

were performed using Seahorse Mito-stress kits (Agilent Technologies, Santa Clara, CA) following the manufacturer's protocol. Data were analyzed using Agilent's Seahorse XF Cell Mito Stress Test Report Generators to summarize and combine the different n values for each group. The summarized values for basal oxygen consumption rate, spare respiratory capacity, proton leak, and ATP production values for each experiment (n) were analyzed in GraphPad (Dotmatics, Boston, MA) using one-way ANOVA and post-hoc Tukey tests.

TRIzol Digestion for Corneal RNA Extraction: After sacrifice, each cornea was placed in 400 µL of TRIzol in 1.5 mL Safe-Lock Tubes with one 100 µL scoop of zirconium oxide (ZO) beads of 1.0 mm diameter and one scoop of ZO beads of 2.5 mm diameter added to each of the tubes. The tubes were then placed in a Next Advanced Bullet Blender Storm Pro at 4°C for 5 minutes at speed 6, followed by a 1-minute rest on ice and then another cycle in the bullet blender for 3 minutes at speed 5. Then the homogenized tissue was brought to 1 mL using TRIzol and incubated for 5 minutes at room temperature. Chloroform (0.2 mL) was added, and the samples were incubated for 3 minutes at room temperature, followed by centrifugation for 5 minutes at 12,000 × g at 4°C. The upper aqueous phase containing RNA was pipetted into separate clean tubes and RNA purified using PureLink RNA Micro kits following the manufacturer's protocol. RT-qPCR was then performed on the purified RNA using the protocol described above.

Supplemental Table S1. Blood glucose levels for diabetic male mice over time: Blood glucose levels were confirmed to be in the range of 249-650 mg/dL at 10 weeks of age by blood collection using the tail snip method and monitoring with an Abbot Freestyle Lite blood glucometer. No difference in blood glucose for the diabetic mouse group treated with or without SAHA was seen for any of 3 different time points as analyzed using Student's T-tests. SAHA was not used until the day of wounding.

		Blood Glucose (mg/dL)		
**Group**	**mouse #**	**Week 1**	**Week 5**	**Week 10**
Diabetes SAHA	311	500	396	455
	312	343	424	361
	313	413	357	396
	314	362	455	480
	315	271	162	231
Diabetes Vehicle	316	367	317	447
	317	304	421	410
	318	271	329	459
	319	298	387	458
	320	382	368	490

A

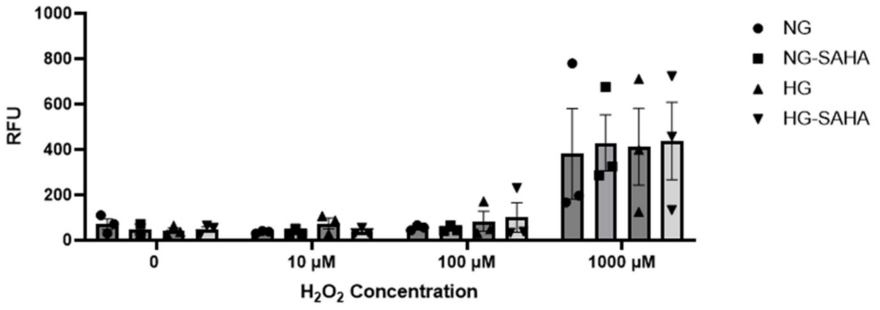

Supplemental Figure S1: Neither glucose status nor SAHA treatment altered ROS levels with or without exposure to hydrogen peroxide as a stressor. HCEC were plated on black 96-well plates at ~75,000 cells/mL. The treatment conditions and timeline followed the plan described in the section on Cell Plating and Treatment Timeline. ROS were measured using the OxiSelect™ Intracellular ROS Assay Kit following the manufacturer's protocol. Briefly, cells were washed 3 times with PBS and incubated with medium without phenol red containing DCFH-DA (0.5 mM) for 40 minutes. The cells were then washed 3 times with PBS and treated with phenol red-free medium containing glucose at 25.0 mM (high glucose or HG) or 5.5 mM (normal glucose or NG, with osmolarity matched with mannitol) with the addition of either 0, 10, 100 or 1000 mM hydrogen peroxide. Fluorescence measurements were recorded on a plate reader at excitation and emission wavelengths of 480 nm and 530 nm, respectively; readings were taken after hydrogen peroxide treatment for 1 hour. This experiment was conducted three separate times and the data analyzed using two-way ANOVA and Tukey post-hoc tests at each concentration of hydrogen peroxide.

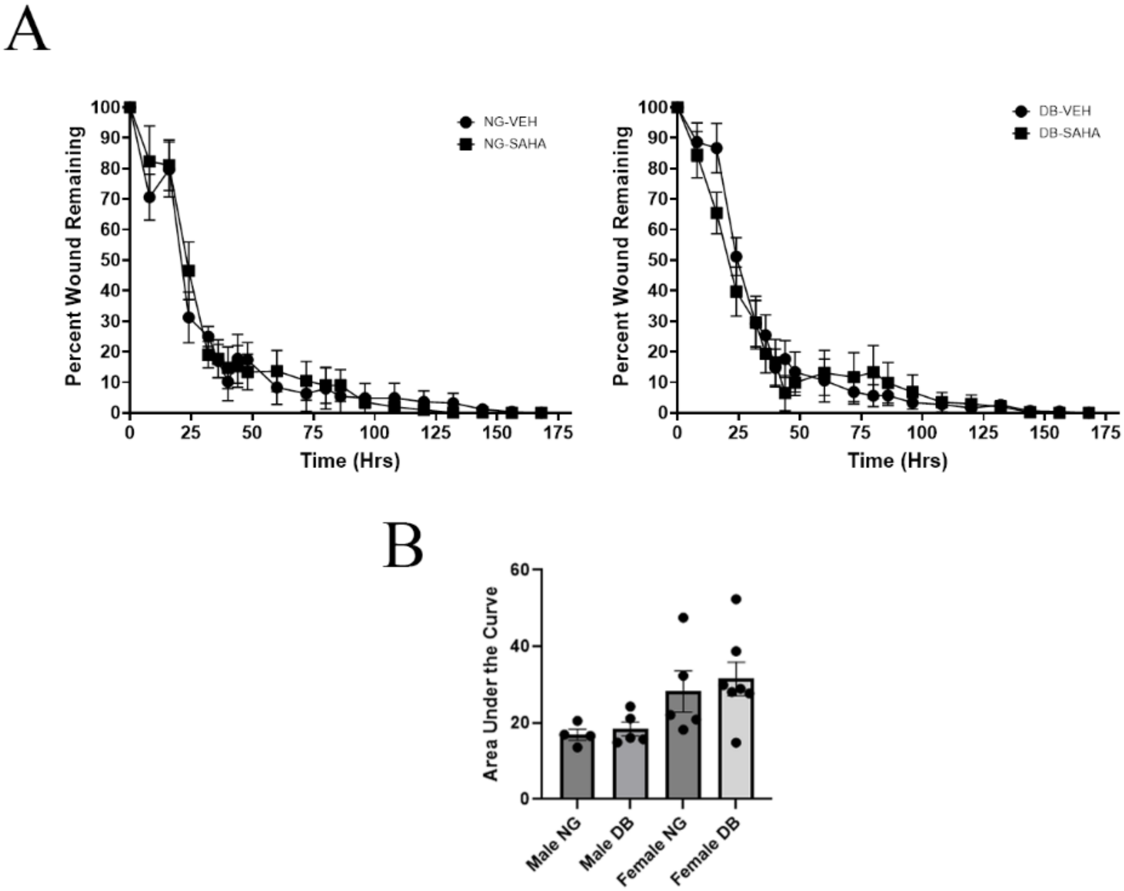

Source	P value
Diabetes	p=0.5707
Sex	p=0.0091
Diabetes*Sex	p=0.8316

Supplemental Figure S2: Female mice healed more slowly than male mice and showed no difference in wound healing regardless of glycemic status and/or treatment with SAHA. A. Female mice showed no difference in wound healing regardless of treatment or glycemic status. C57BL/6 female mice treated with a drop of 10μm SAHA or PBS (Vehicle control) every 4 hours after wounding were anesthetized with isoflurane, and the wound visualized using fluorescein to monitor healing; no difference in wound healing rates was observed. Results are shown as mean ± SEM and analyzed by Student's t-test at each time point (n=4-5). **B. Female mice healed more slowly than male mice regardless of glycemic status.** The areas under the curve for normoglycemic and diabetic mice (diabetic for 10 weeks) for both male (20 weeks old) and female (21 weeks old) were analyzed by two-way ANOVA (table results are shown under the graph) and demonstrated that female mice healed more slowly than male mice irrespective of glycemic status (n=4-6). NG=Normoglycemic; DB=Diabetic.

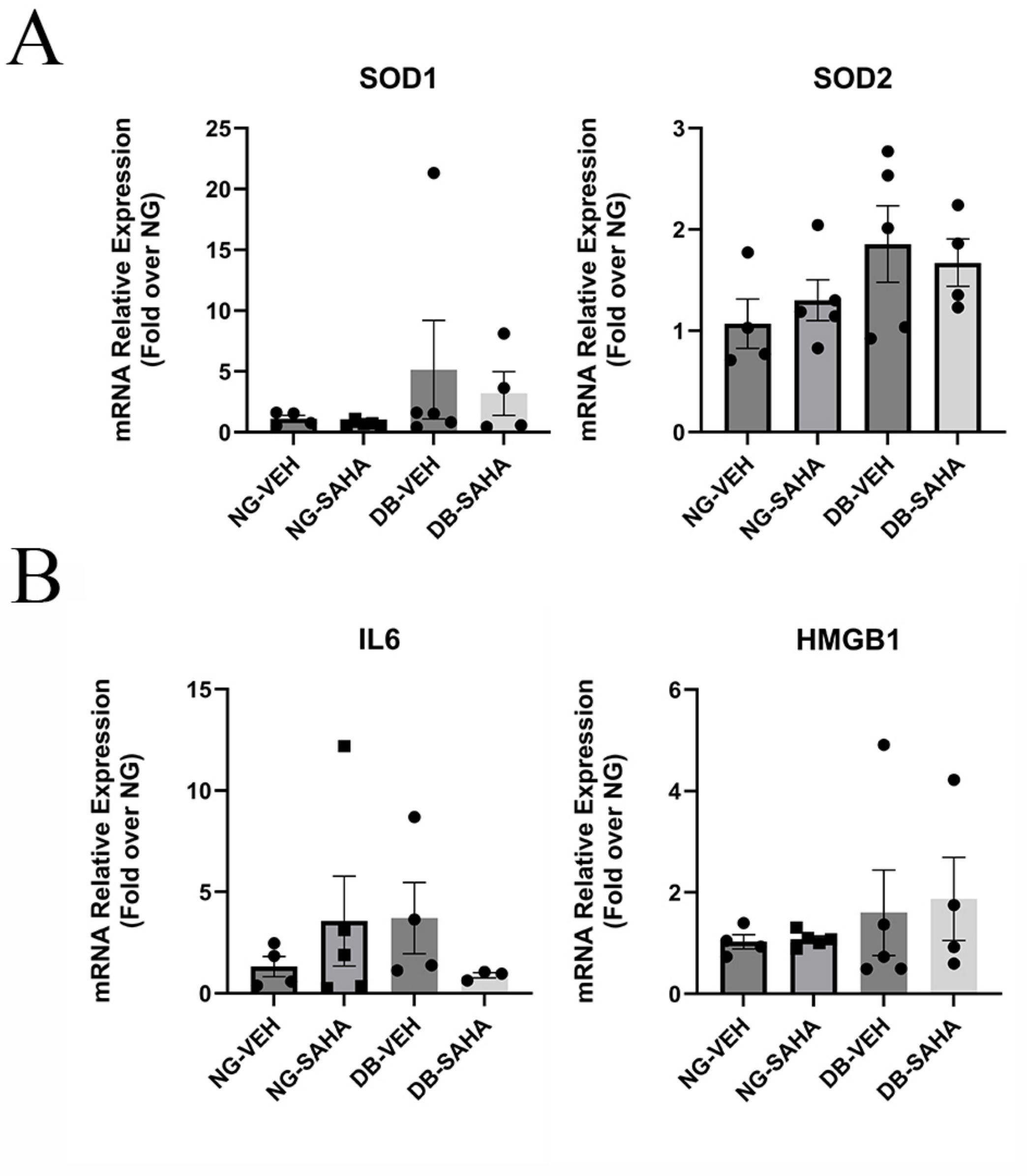

Supplemental Figure S3: ROS Scavenger genes Sod1 and Sod2, damage-associated molecular pattern Hmgb1, and inflammatory molecule Il6, exhibited no change in expression regardless of diabetes or SAHA treatment. (A). Healed corneas collected from diabetic and normoglycemic mice treated with or without SAHA were collected and digested with Trizol for RNA extraction for RT-qPCR. Analysis was performed by two-way ANOVA and post hoc Tukey tests on delta-Ct values (n = 4–5). NG = normoglycemic; DB = diabetic; VEH = vehicle; SAHA = SAHA (10 μM). (B). RNA was isolated from healed corneas collected from diabetic and normoglycemic mice treated with or without SAHA as described above. Results are shown as means ± SEM. Analysis was performed by two-way ANOVA and post hoc Tukey tests as above (n = 4–5). NG = normoglycemic; DB = diabetic; VEH = vehicle; SAHA = SAHA (10 μM).
